# MBNL1 reverses the proliferation defect of skeletal muscle satellite cells in myotonic dystrophy type 1 by inhibiting autophagy via the mTOR pathway

**DOI:** 10.1038/s41419-020-02756-8

**Published:** 2020-07-18

**Authors:** Kai-Yi Song, Xiu-Ming Guo, Hui-Qi Wang, Lei Zhang, Si-Yuan Huang, Ying-Chao Huo, Gang Zhang, Jin-Zhou Feng, Rong-Rong Zhang, Yue Ma, Qing-Zhe Hu, Xin-Yue Qin

**Affiliations:** 1 Department of Neurology, The First Affiliated Hospital of Chongqing Medical University, 1 Yixueyuan Road, Yuzhong District, 400016 Chongqing, China; 2 Rehabilitation & Sports Medicine Research Institute of Zhejiang Province, Zhejiang Provincial People’s Hospital, People’s Hospital of Hangzhou Medical College, 310014 Hangzhou, China; 3 Department of Cerebrovascular Diseases, The first people’s hospital of Zunyi, The third affiliate hospital of Zunyi medical university, Number 98, Fenghuang Road, 563000 Zunyi, Guizhou Province China

**Keywords:** Mechanisms of disease, Induced pluripotent stem cells

## Abstract

Skeletal muscle atrophy is one of the clinical symptoms of myotonic dystrophy type 1 (DM1). A decline in skeletal muscle regeneration is an important contributor to muscle atrophy. Skeletal muscle satellite cells (SSCs) drive skeletal muscle regeneration. Increased autophagy can reduce the proliferative capacity of SSCs, which plays an important role in the early regeneration of damaged skeletal muscle in DM1. Discovering new ways to restore SSC proliferation may aid in the identification of new therapeutic targets for the treatment of skeletal muscle atrophy in DM1. In the pathogenesis of DM1, muscleblind-like 1 (MBNL1) protein is generally considered to form nuclear RNA foci and disturb the RNA-splicing function. However, the role of MBNL1 in SSC proliferation in DM1 has not been reported. In this study, we obtained SSCs differentiated from normal DM1-04-induced pluripotent stem cells (iPSCs), DM1-03 iPSCs, and DM1-13-3 iPSCs edited by transcription activator-like (TAL) effector nucleases (TALENs) targeting CTG repeats, and primary SSCs to study the pathogenesis of DM1. DM1 SSC lines and primary SSCs showed decreased MBNL1 expression and elevated autophagy levels. However, DM1 SSCs edited by TALENs showed increased cytoplasmic distribution of MBNL1, reduced levels of autophagy, increased levels of phosphorylated mammalian target of rapamycin (mTOR), and improved proliferation rates. In addition, we confirmed that after MBNL1 overexpression, the proliferative capability of DM1 SSCs and the level of phosphorylated mTOR were enhanced, while the autophagy levels were decreased. Our data also demonstrated that the proliferative capability of DM1 SSCs was enhanced after autophagy was inhibited by overexpressing mTOR. Finally, treatment with rapamycin (an mTOR inhibitor) was shown to abolish the increased proliferation capability of DM1 SSCs due to MBNL1 overexpression. Taken together, these data suggest that MBNL1 reverses the proliferation defect of SSCs in DM1 by inhibiting autophagy via the mTOR pathway.

## Introduction

Myotonic dystrophy type 1 (DM1) is a neuromuscular disease caused by expanded CTG repeats in the myotonic dystrophy protein kinase (DMPK) gene^1,2^. The mutant DMPK gene is also transcribed into CUGn-containing RNA, which forms nuclear RNA foci and disturbs RNA-binding proteins, resulting in reduced levels of MBNL1 and increased levels of CUG-binding protein 1 (CUGBP1)^3^. Muscle atrophy is one of the core symptoms of DM1^1,4^, with decreased skeletal muscle regeneration as an important contributor^5^. New methods of enhancing the impaired regeneration of skeletal muscle are essential to improve skeletal muscle atrophy and the quality of life of DM1 patients.

Skeletal muscle satellite cells (SSCs) are the source of skeletal muscle regeneration. Increased SSC proliferation can promote the early regeneration of skeletal muscle^6^. SSCs are often located between the basal lamina and the plasma membrane in muscle fibers^7^. SSCs are at rest under normal conditions, but when muscles are damaged, they become activated and proliferate to form new skeletal muscle fibers^6,8^. However, the SSC proliferation capacity of DM1 patients is impaired^9^. The basic mechanisms of this process are not fully understood. Therefore, seeking ways to increase the proliferation of SSCs could inform the treatment of muscular atrophy.

The MBNL1 protein is an important factor responsible for RNA splicing^10^. In DM1, the function of the MBNL1 protein is dysregulated due to the binding of MBNL1 to CUG expansion (CUGexp) RNA, which forms intranuclear lesions^11^. Previous studies have also shown that MBNL1 may be involved in the regulation of muscle atrophy in Drosophila models of DM2^12,13^. In addition, atrophic muscle fibers are found in MBNL1-knockout mice^14^, but whether this is caused by affecting SSC proliferation activity has not been investigated in DM1. One study showed that DM1 neural stem cells exhibit defects in proliferation and increased autophagy associated with changes in the mTOR pathway. Furthermore, loss of MBNL1 function leads to changes in the mTOR pathway, and gain of MBNL1 function rescues the disease phenotype, including autophagy-induced reduction. The study also suggested that autophagy induction may also be involved in the pathogenesis of DM1^15^. Notably, mTOR is an atypical serine/threonine protein kinase that acts as an important regulator of skeletal muscle atrophy and regeneration^16^. Some studies have demonstrated that mTOR can promote the proliferation of primary SSCs^17^. Therefore, MBNL1 may regulate the proliferation of SSCs via the mTOR pathway.

Autophagy, also known as “self-eating,” is a process involving the degradation of proteins and organelles to recycle substances in response to cellular stress^18^. Autophagy plays a role in various pathophysiological events, including cell proliferation, metabolic abnormalities, and cell differentiation, death, and growth^19,20^. However, macrophagy (henceforth named autophagy), a protein-degradation system closely related to cell proliferation^21^, is also involved in repairing damaged skeletal muscle^22^ and reversing muscle atrophy^23,24^. Previous studies have reported that melatonin can promote proliferation of neural stem cells by repressing elevated autophagy under hyperglycemic conditions^25^. Suppressing autophagy can also enhance the proliferation of germ cells^26^. Notably, mTOR is a major regulator of cell autophagy and proliferation^19,20^. In addition, overexpression of TOR (the human homolog of mTOR) inhibited autophagy to ameliorate atrophy of indirect flight muscles in a Drosophila model of DM1^27^. Furthermore, MBNL1 modulated the mTOR pathway^15^. Therefore, MBNL1 may promote proliferation of DM1 SSCs by affecting autophagy levels via the mTOR pathway.

Studies investigating the pathogenesis of DM1 usually rely on mouse models, Drosophila models^28^. Muscle biopsy is also an effective method to obtain SSCs to describe this pathogenesis^29^. However, it causes pain to the patient and yields a limited number of SSCs^30^. In contrast, patient-derived induced pluripotent stem cells (iPSCs) not only carry the patient’s disease-causing genetic information, but can also be robustly differentiated into SSCs; thus, iPSCs are important tools for research on the molecular and functional abnormalities of DM1^31^. Currently, we can obtain iPSCs from reprogrammed somatic cells and establish patient-specific cell models to study the molecular and functional abnormalities of human diseases^32^. We obtained DM1-03 iPSCs derived from a DM1 patient and normal DM1-04 iPSCs from a healthy volunteer. DM1-03 iPSCs contain 2829–3575 CTG repeats^33^. We have also developed a method for the directional insertion of polyA signals (PASs) upstream of the DMPK CTG repeats in DM1-03 iPSCs, which eliminates nuclear RNA foci and reverses the phenotype of MBNL1. We named genetically edited DM1-13-3 iPSCs. Furthermore, all three types of iPSCs maintain their pluripotency^33,34^.

Here, we use SSCs differentiated from DM1 iPSCs and DM1 primary SSCs to study the mechanism responsible for the disruption of SSC proliferation in DM1. We named the corresponding SSCs from DM1-04 iPSCs, DM1-03 iPSCs, and DM1-13-3 iPSCs DM1-04, DM1-03, and DM1-13-3. Together, we innovatively obtained SSCs differentiated from DM1 iPSCs and used them as cellular models to study the pathogenesis of DM1, although we were unable to determine the role of MBNL1 in DM1 SSCs. Therefore, this study mainly investigated whether MBNL1 reverses the proliferation defect of DM1 SSCs by inhibiting autophagy via the mTOR pathway.

## Results

### DM1 SSCs exhibit a proliferation defect, and the TALEN genome modification can ameliorate the proliferation defect

**Fig. 1:**
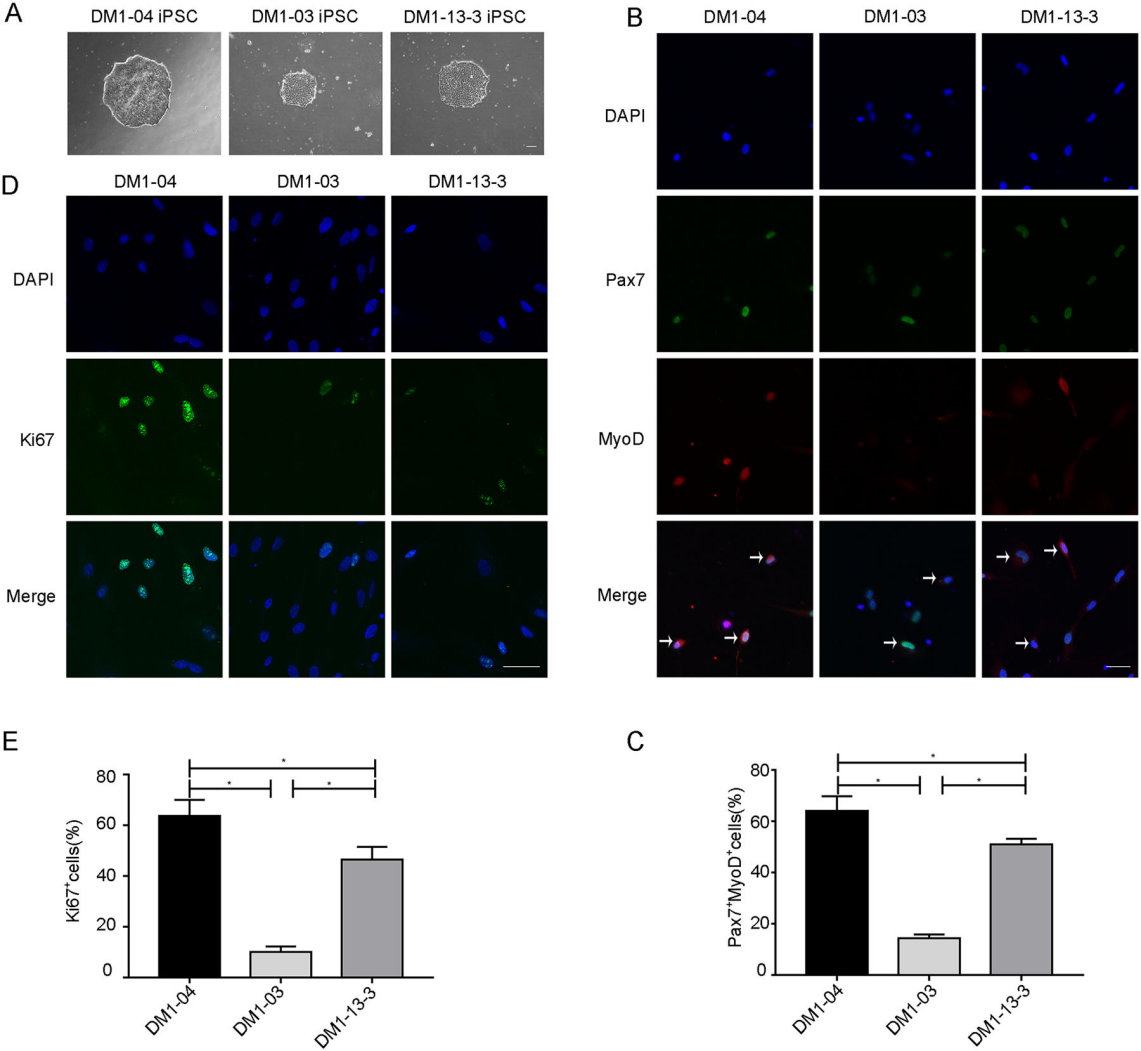
The proliferative capacity of DM1 SSCs is impaired. **a** Morphology of the three cell lines (scale bar = 100 μm). **b** Representative immunofluorescence images of Pax7 (green), MyoD (red), and DAPI (blue) (scale bar = 50 μm) (*n* = 3). Arrows show the positive cells. **c** The proportion of proliferating SSCs (Pax7^+^MyoD^+^) in three cell lines (*n* = 3). **d**, **e** Proliferation was evaluated in three cell lines by immunofluorescence for Ki67 (green). Nuclei were stained with DAPI (blue) (*n* = 3). Scale bar = 50 μm. The bars represent the means ± SD; one-way ANOVA with Bonferroni post hoc test, *P < 0.05.

There were no differences in morphology among the three iPSC lines (Fig. 1a), all of which successfully differentiated into SSCs (DM1-04, DM1-03, and DM1-13-3) and expressed the cell markers Pax7 and MyoD (Fig. 1b). To explore the proliferation ability of DM1 SSCs, we performed Pax7, MyoD, and Ki67 immunofluorescence. The proportion of proliferating SSCs (Pax7^+^MyoD^+^) in the DM1-03 group was lower than that in the DM1-04 group, while that in the DM1-13-3 group was higher than that in the DM1-03 group, but was lower than that in the DM1-04 group (Fig. 1b, c). Ki67 immunofluorescence showed that the proportion of Ki67-positive cells in the DM1-03 group was lower than that in the DM1-04 group, while the proportion in the DM1-13-3 group was higher than that in the DM1-03 group but lower than that in the DM1-04 group (Fig. 1d, e). These results suggest that DM1 SSCs have proliferation defects, and TALEN genome modification can ameliorate the proliferation defect although it remains lower than normal.

### The TALEN genome modification eliminates nuclear RNA foci and redistributes and enhances MBNL1 protein expression

**Fig. 2:**
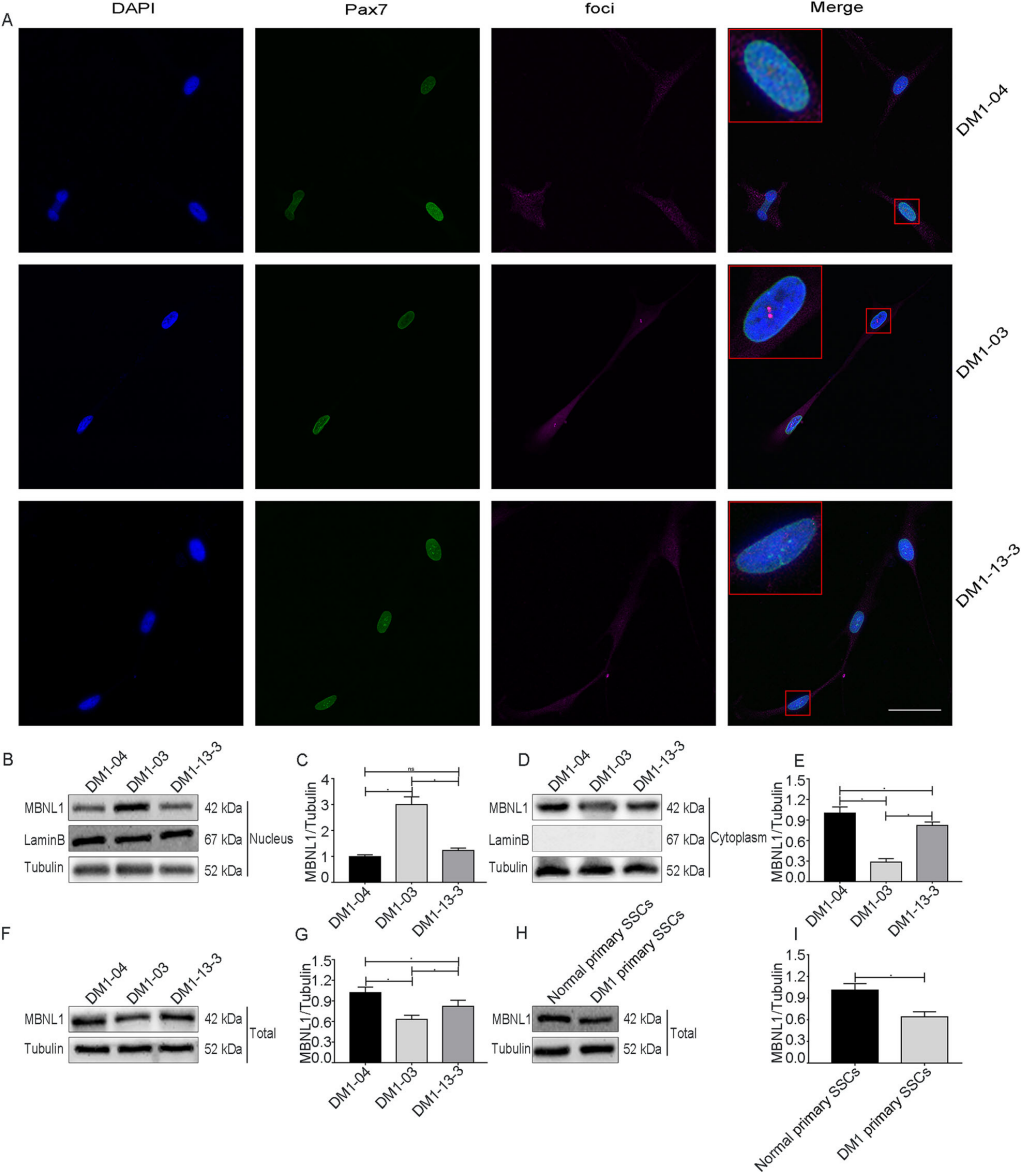
TALEN genome modification eliminates nuclear foci and increases cytoplasmic and total MBNL1 expression in DM1 SSCs. **a** Representative images of RNA fluorescence in situ hybridization for Pax7 (green), a Cy5-labeled (CAG)10 DNA probe (Cy5), and DAPI (blue) in three cell lines (scale bar = 50 μm) (*n* = 3). Insets show magnified images of representative cells. **b**, **c** Representative images of nuclear MBNL1 expression in the three SSC lines (*n* = 3). **d**, **e** Representative images of cytoplasmic MBNL1 expression in three SSC lines (*n* = 3). **f**, **g** Representative images of total MBNL1 expression in three SSC lines (*n* = 3). **h**, **i** Representative images of total MBNL1 expression in primary SSCs (*n* = 3). The bars represent the means ± SD; Student’s test or one-way ANOVA with Bonferroni post hoc test, *P < 0.05.

To explore the effect of TALEN genome modification on the lesions in the nucleus of DM1 SSCs, we performed RNA fluorescence in situ hybridization. First, we used a Cy5-labeled (CAG)10 DNA probe to detect nuclear RNA foci, and found that nuclear RNA foci were present in DM1-03 SSCs (Fig. 2a). However, DM1-13-3 SSCs were all devoid of nuclear RNA foci using this method (Fig. 2a). Furthermore, we detected the nuclear, cytoplasmic, and total expression of MBNL1 protein in DM1 SSCs by western blot analysis. Nuclear MBNL1 protein expression in DM1-03 SSCs was higher than that in DM1-04 SSCs (Fig. 2b, c), while that in DM1-13-3 SSCs was lower than that in DM1-03 SSCs (Fig. 2b, c). However, no significant difference was found between the DM1-04 and DM1-13-3 SSCs (Fig. 2b, c). The cytoplasmic and total expression of MBNL1 protein in DM1-03 SSCs was significantly lower than that in DM1-04 SSCs, while that in DM1-13-3 SSCs was higher than that in DM1-03 SSCs, but lower than that in DM1-04 SSCs (Fig. 2d–g). Likewise, the total expression of MBNL1 protein in DM1 primary SSCs was significantly lower than in normal primary SSCs from healthy volunteers (Fig. 2h, i). These results suggest that total and cytoplasmic MBNL1 protein is decreased in DM1 SSCs. TALEN genome modification eliminates nuclear RNA foci and redistributes and enhances MBNL1 protein expression. In addition, it can promote the proliferation of DM1 SSCs. Therefore, MBNL1 may regulate the proliferation capacity of DM1 SSCs.

### MBNL1 overexpression promotes the proliferative capacity of DM1 SSCs

**Fig. 3:**
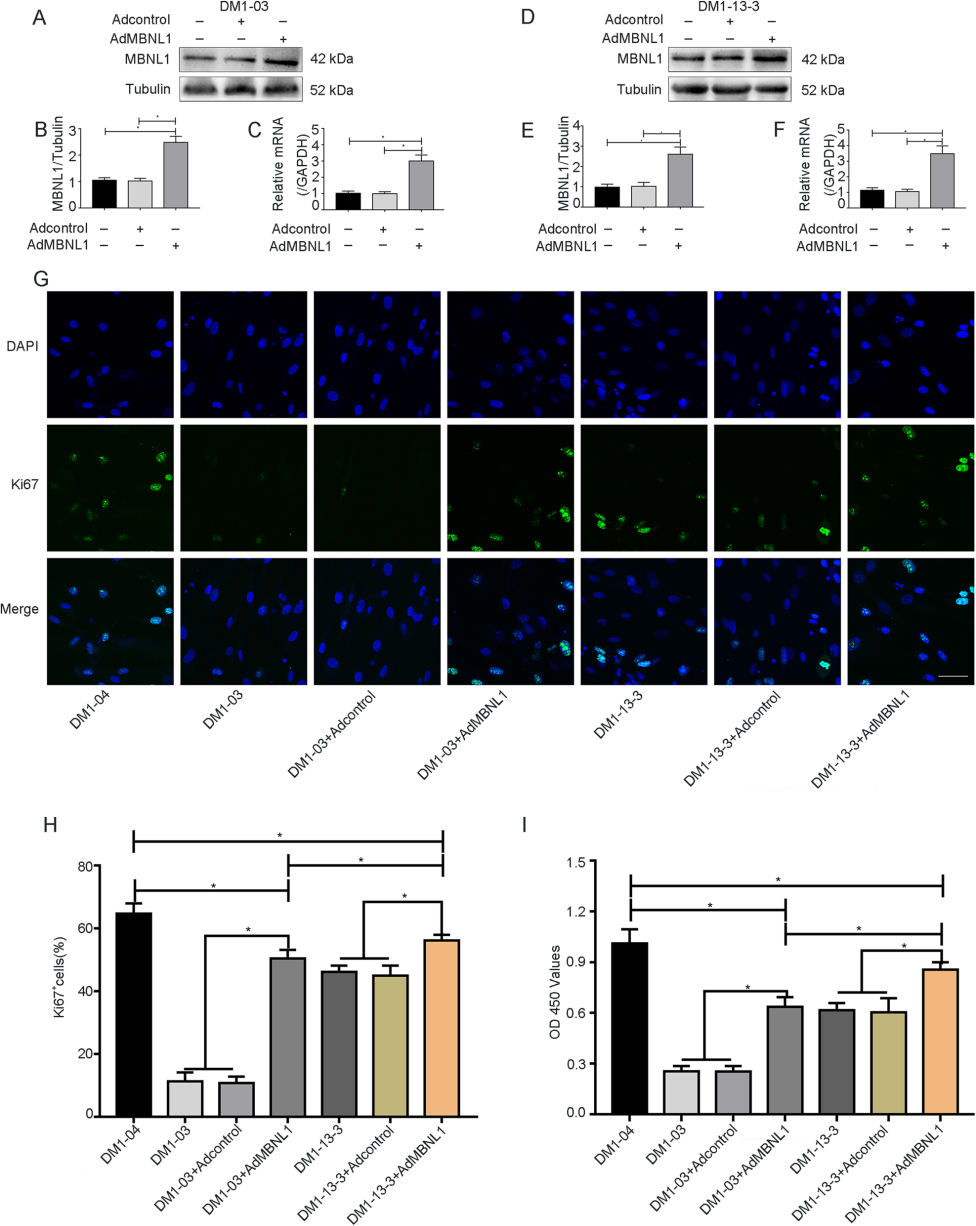
The proliferative capacity of DM1-03 and DM1-13-3 SSCs increased after MBNL1 overexpression. **a**–**c** MBNL1 expression in DM1-03 was increased at the protein and mRNA levels after MBNL1 was overexpressed (*n* = 3). **d**–**f** MBNL1 expression in DM1-13-3 was increased at the protein and mRNA levels after MBNL1 was overexpressed (*n* = 3). **g**, **h** Proliferation in DM1-03 and DM1-13-3 after MBNL1 overexpression was evaluated by immunofluorescence for Ki67 (green); nuclei were stained with DAPI (blue) (scale bar = 50 μm) (*n* = 3). **i** Proliferation in DM1-03 and DM1-13-3 after MBNL1 overexpression was evaluated with CCK-8 assays (*n* = 3). The bars represent the means ± SD; one-way or two-way ANOVA with Bonferroni post hoc test, *P < 0.05.

To investigate the role of MBNL1 in the proliferation of DM1 SSCs, we conducted adenoviral overexpression of MBNL1 in DM1-03 and DM1-13-3 SSCs. Significantly elevated protein and mRNA expression levels of MBNL1 in the DM1-03 and DM1-13-3 SSCs were detected by western blot analysis and qRT-PCR (Fig. 3a–f). To examine the proliferative capacity of DM1 SSCs after MBNL1 overexpression, we conducted Ki67 immunofluorescence and CCK-8 assays. Ki67 immunofluorescence showed that the proportion of Ki67-positive cells in DM1-03 and DM1-13-3 groups was enhanced after MBNL1 overexpression, but was still lower than that in the DM1-04 group (Fig. 3g, h). The proportion of Ki67-positive cells in DM1-13-3+Ad-MBNL1 was higher than that in DM1-03+Ad-MBNL1 (Fig. 3g, h). Likewise, similar results were observed in DM1 SSCs with CCK-8 assays (Fig. 3i). Hence, MBNL1 can promote the proliferation capacity of DM1 SSCs, and this effect was more pronounced in the genome-modification group.

### The TALEN genome modification can reduce the abnormally elevated autophagy levels in DM1 SSCs

**Fig. 4:**
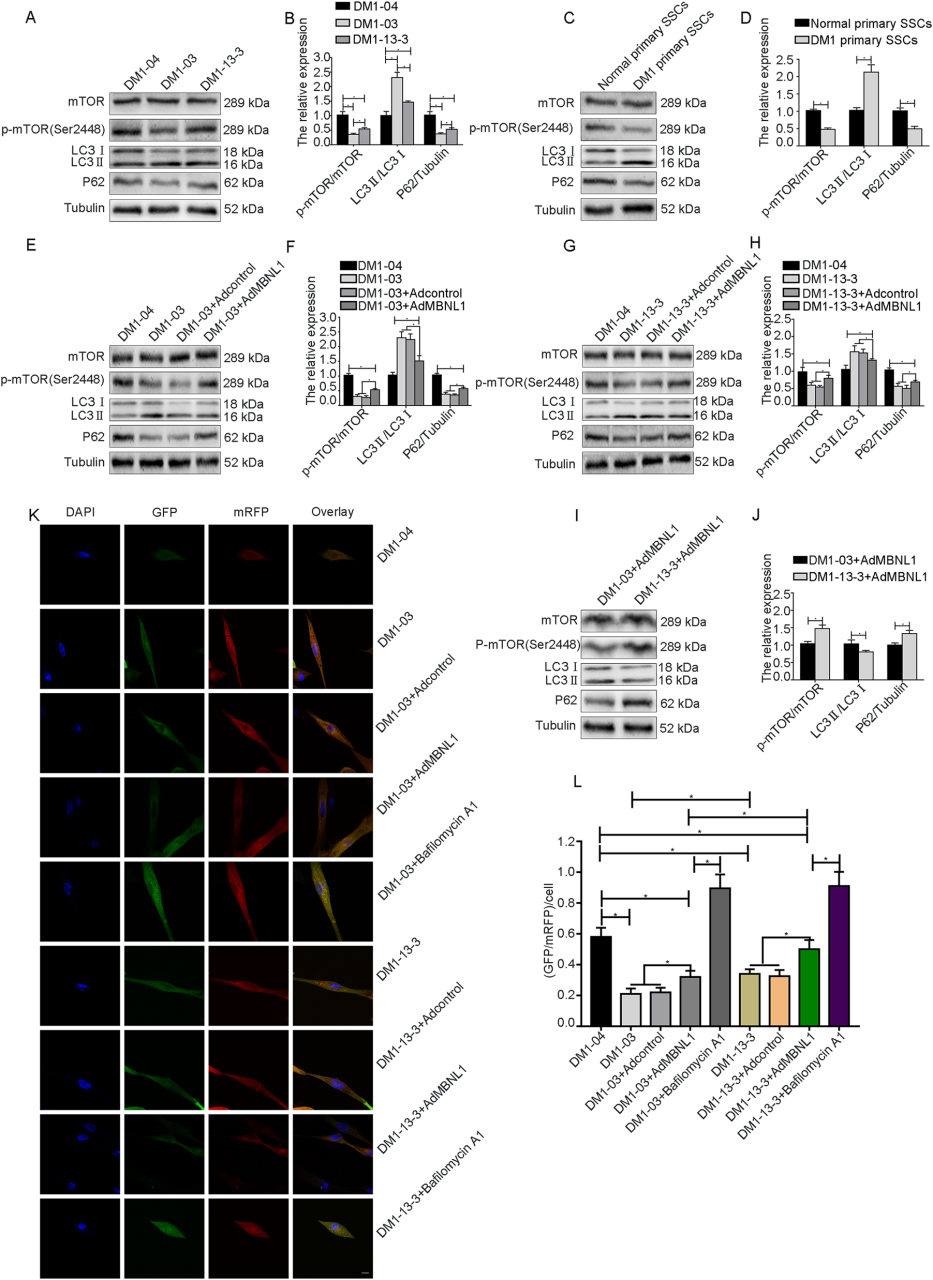
Overexpression of MBNL1 reduces elevated autophagy levels in DM1 SSCs. **a**–**d** The autophagy levels in DM1-03, DM1-13-3, and DM1 primary SSCs were evaluated by western blot analysis (*n* = 3). **e**–**j** The expression of p-mTOR/mTOR, LC3II/LC3I, and P62 in DM1 SSCs after overexpression of MBNL1 was detected by western blot analysis (*n* = 3). **k**, **l** The autophagy levels in DM1 SSCs after MBNL1 was overexpressed were evaluated by immunofluorescence for GFP (green), mRFP (red), and DAPI (blue) (*n* = 3). Scale bar = 10 μm. The bars represent the means ± SD; Student’s test or ANOVA with Bonferroni post hoc test, *P < 0.05.

We next performed western blot analysis and GFP-mRFP-LC3 to detect the autophagy levels in the DM1-04, DM1-03, and DM1-13-3 groups. Western blot analysis was also conducted to detect the autophagy levels in primary SSCs from healthy volunteers and DM1 patients. The ratio of LC3II/LC3I in DM1-03 SSCs was significantly higher than that in DM1-04 SSCs, while that in DM1-13-3 SSCs was significantly lower than that in DM1-03 SSCs, but higher than that in DM1-04 SSCs (Fig. 4a, b). The levels of p-mTOR/mTOR and P62 in DM1-03 SSCs were significantly lower than those in DM1-04 SSCs, while those in DM1-13-3 SSCs were significantly higher than those in DM1-03 SSCs, but lower than those in DM1-04 SSCs (Fig. 4a, b). Similar results were observed in primary SSCs from healthy volunteers and DM1 patients (Fig. 4c, d). In addition, after treatment with GFP-mRFP-LC3, the GFP/mRFP per cell in DM1-03 SSCs was significantly lower than that in DM1-04 SSCs, while that in DM1-13-3 SSCs was higher than that in DM1-03 SSCs, but lower than that in DM1-04 SSCs (Fig. 4k, l). Thus, the autophagy levels in DM1 SSCs are increased, and TALEN genome modification can reduce the abnormal autophagy levels in DM1 SSCs.

### MBNL1 overexpression enhances the level of phosphorylated mTOR, while it decreases autophagic flux in DM1 SSCs

To investigate the role of MBNL1 in the mTOR pathway and autophagy in DM1 SSCs, we performed western blot analysis and GFP-mRFP-LC3. According to the western blot results, the levels of p-mTOR/mTOR and P62 in DM1 SSCs were increased after MBNL1 overexpression, but were still lower than those in the DM1-04 group (Fig. 4e–h). The LC3II/LC3I ratio in DM1 SSCs decreased after MBNL1 overexpression, but was still higher than that in the DM1-04 group (Fig. 4e–h). The levels of p-mTOR/mTOR and P62 in DM1-13-3+Ad-MBNL1 SSCs were higher than those in DM1-03+Ad-MBNL1 SSCs, whereas the LC3II/LC3I ratio in DM1-13-3+Ad-MBNL1 SSCs was lower than that in DM1-03+Ad-MBNL1 SSCs (Fig. 4i, j).

The analysis of GFP-mRFP-LC3 showed that the GFP/mRFP per cell in DM1 SSCs was increased after MBNL1 overexpression, but was still lower than that in DM1-04 SSCs (Fig. 4k, l). In addition, the GFP/mRFP per cell in DM1-13-3+Ad-MBNL1 SSCs was higher than that in DM1-03+Ad-MBNL1 SSCs (Fig. 4k, l). MBNL1 significantly inhibited the autophagic flux in DM1 SSCs, but this inhibitory effect was not as obvious as that of Bafilomycin A1 (Fig. 4k, l). Together, MBNL1 can enhance the level of phosphorylated mTOR and reduce autophagy induction in DM1 SSCs, and this effect was more pronounced in the genome-modification group.

### Inhibiting autophagy activation promotes DM1 SSC proliferation

To further explore the role of autophagy activation in DM1 SSC proliferation, we conducted western blot analysis, CCK-8 assays, and Ki67 immunofluorescence to evaluate the proliferation of DM1 SSCs after autophagy was suppressed by mTOR overexpression.

**Fig. 5:**
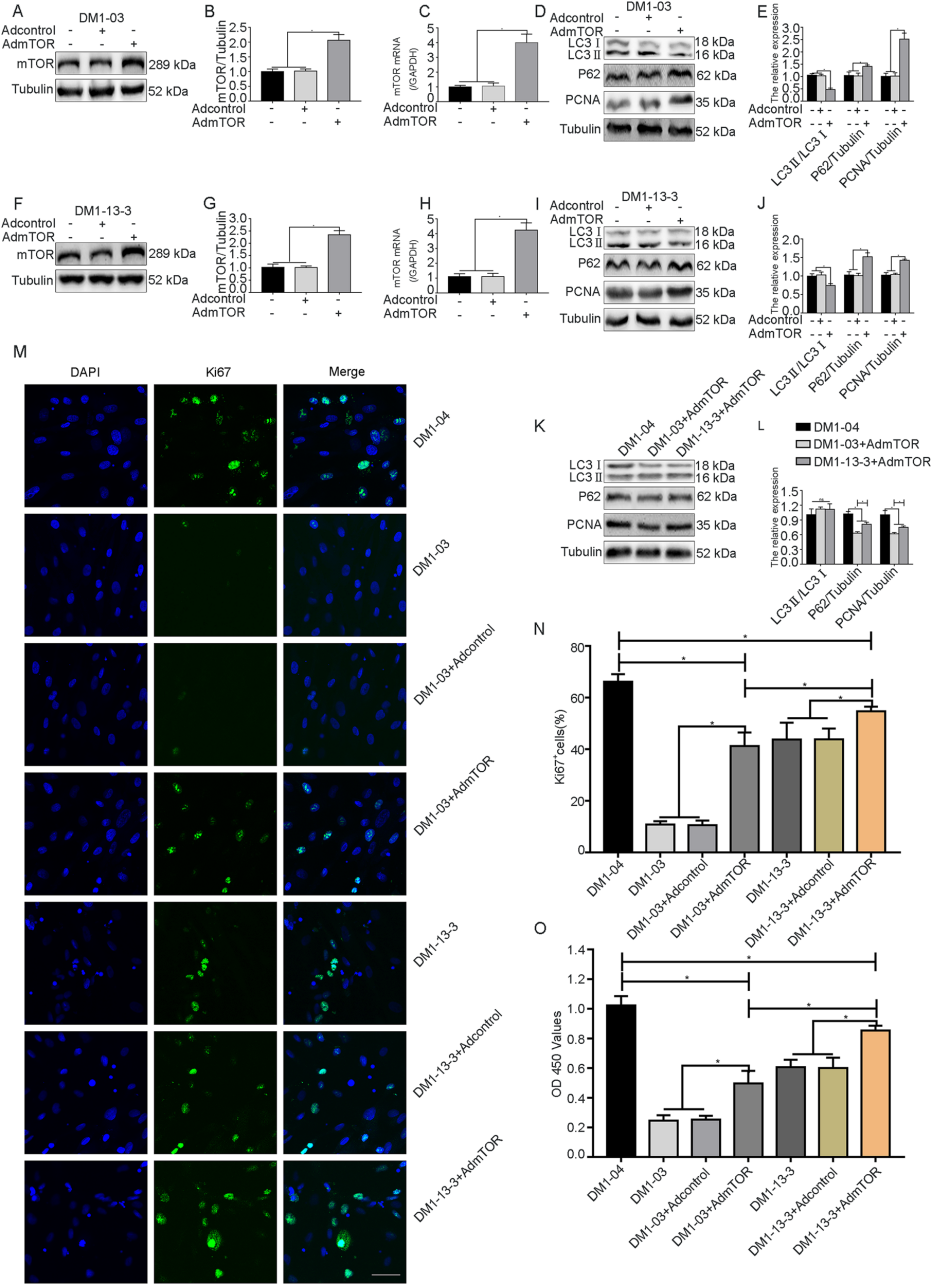
Inhibition of autophagy can promote DM1 SSC proliferation. **a**–**c**, **f**–**h** The expression of mTOR in DM1-03 and DM1-13-3 SSCs was increased at the protein and mRNA levels after mTOR overexpression (*n* = 3). **d**, **e**, **i**–**l** The expression of LC3II/LC3I, P62, and PCNA in DM1 SSCs after mTOR overexpression was evaluated by western blot analysis (*n* = 3). **m**, **n** Proliferation in DM1 SSCs after mTOR overexpression was evaluated with immunofluorescence for Ki67 (green). Nuclei were stained with DAPI (blue). Scale bar = 50 μm. (*n* = 3). **o** Proliferation in DM1 SSCs after mTOR overexpression was evaluated with CCK-8 assays (*n* = 3). The bars represent the means ± SD; one-way or two-way ANOVA with Bonferroni post hoc test, *P < 0.05.

The protein and mRNA expression levels of mTOR in DM1-03 (Fig. 5a–c) and DM1-13-3 SSCs (Fig. 5f–h) were significantly enhanced after its overexpression. Western blot results showed that the levels of LC3II/LC3I in DM1-03 (Fig. 5d, e) and DM1-13-3 SSCs (Fig. 5i, j) decreased after mTOR overexpression, and there were no differences from DM1-04 SSCs (Fig. 5k, l). The expression of P62 and proliferating cell nuclear antigen (PCNA) in DM1-03 (Fig. 5d, e) and DM1-13-3 SSCs (Fig. 5i, j) increased after mTOR overexpression, but was still lower than that in DM1-04 SSCs (Fig. 5k, l). There was no difference in LC3II/LC3I between DM1-03+Ad-mTOR and DM1-13-3+Ad-mTOR SSCs (Fig. 5k, l). The expression of PCNA and P62 in DM1-13-3+Ad-mTOR SSCs was higher than that in DM1-03+Ad-mTOR SSCs (Fig. 5k, l).

Ki67 immunofluorescence showed that the proportion of Ki67-positive DM1 SSCs was enhanced after mTOR overexpression, but the proportion was still lower than that in DM1-04 SSCs (Fig. 5m, n). The proportion of Ki67-positive cells in the DM1-13-3+Ad-mTOR group was higher than that in the DM1-03+Ad-mTOR group (Fig. 5m, n). Similar results were observed in SSCs with CCK-8 assays (Fig. 5o).

These results suggest that inhibiting autophagy activation by mTOR overexpression promotes DM1 SSC proliferation, and this effect was more pronounced in the genome-modification group.

### MBNL1 overexpression enhances DM1 SSC proliferation by suppressing autophagy via the mTOR pathway

To investigate whether MBNL1 promotes proliferation by inhibiting autophagy via the mTOR pathway in DM1 SSCs, we performed western blot analysis, CCK-8 assays, and Ki67 immunofluorescence to examine the proliferation of DM1-03+Ad-MBNL1 and DM1-13-3+Ad-MBNL1 SSCs after rapamycin treatment.

**Fig. 6:**
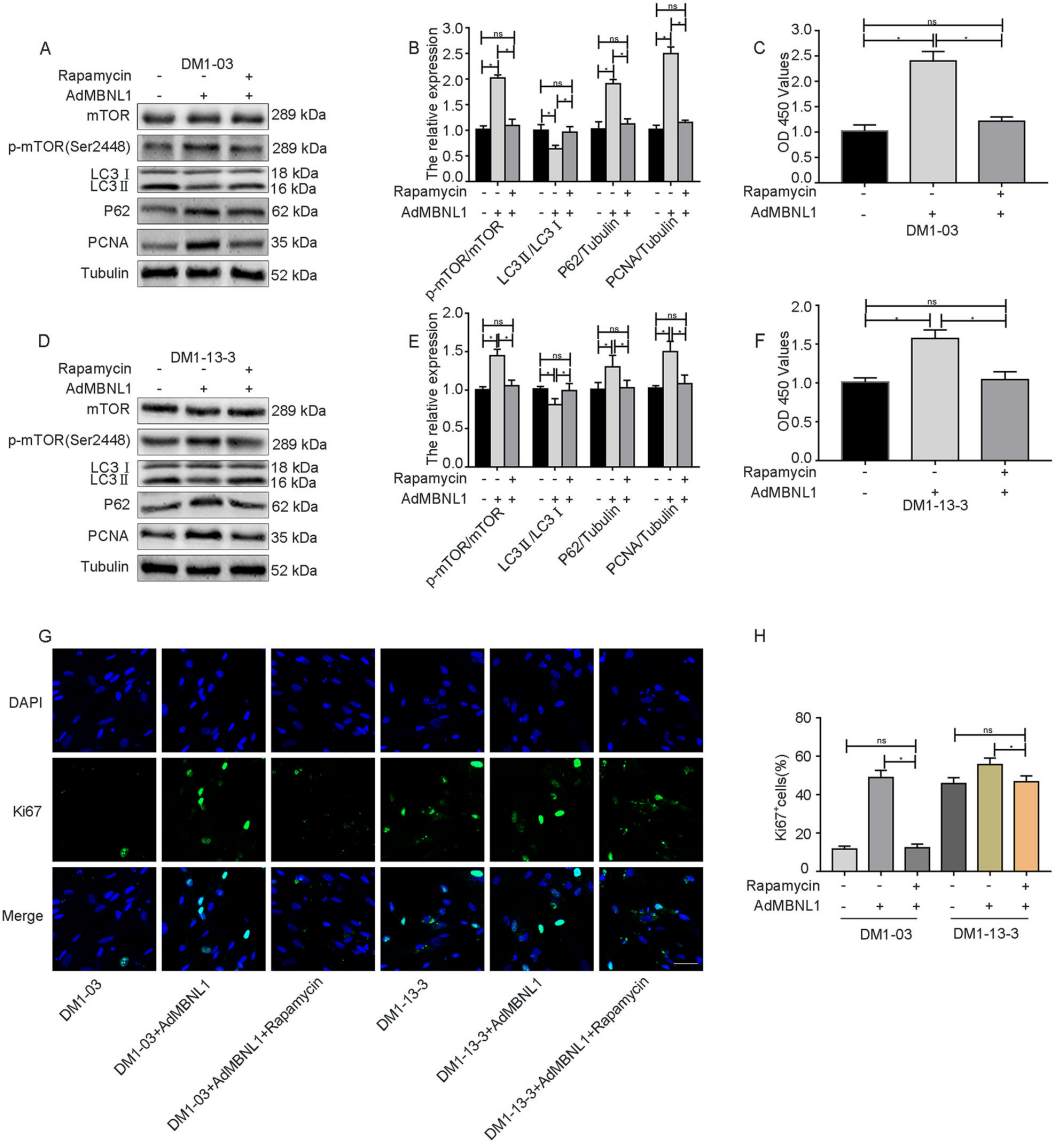
The improved proliferative capacity due to MBNL1 overexpression was abolished after treatment with rapamycin in DM1 SSCs. **a**, **b** The levels of p-mTOR/mTOR, LC3II/LC3I, P62, and PCNA in the DM1-03, DM1-03+Ad-MBNL1, and DM1-03+Ad-MBNL1+rapamycin groups were evaluated by western blot analysis (*n* = 3). **c** Proliferation in the DM1-03, DM1-03+Ad-MBNL1, and DM1-03+Ad-MBNL1+rapamycin groups was evaluated with CCK-8 assays (*n* = 3). **d**, **e** The levels of p-mTOR/mTOR, LC3II/LC3I, P62, and PCNA in the DM1-13-3, DM1-13-3+Ad-MBNL1, and DM1-13-3+Ad-MBNL1+rapamycin groups were evaluated by western blot analysis (*n* = 3). **f** Proliferation in the DM1-13-3, DM1-13-3+Ad-MBNL1, and DM1-13-3+Ad-MBNL1+rapamycin groups was evaluated with CCK-8 assays (*n* = 3). **g**, **h** Proliferation in the DM1-03, DM1-03+Ad-MBNL1, DM1-03+Ad-MBNL1+rapamycin, DM1-13-3, DM1-13-3+Ad-MBNL1, and DM1-13-3+Ad-MBNL1+rapamycin groups was evaluated with Ki67 immunofluorescence (*n* = 3). The bars represent the means ± SD; one-way ANOVA with Bonferroni post hoc test, *P < 0.05.

According to the western blot results, the level of LC3II/LC3I in DM1-03+Ad-MBNL1 (Fig. 6a, b) was increased after rapamycin treatment. The levels of p-mTOR/mTOR, PCNA, and P62 in DM1-03+Ad-MBNL1 (Fig. 6a, b) were significantly reduced after rapamycin treatment. There were no significant differences in the levels of LC3II/LC3I, p-mTOR/mTOR, PCNA, and P62 between the DM1-03+Ad-MBNL1+rapamycin and DM1-03 groups (Fig. 6a, b). Similar results were observed in DM1-13-3 SSCs (Fig. 6d, e).

Both OD 450 values and the proportion of Ki67-positive cells in DM1-03+Ad-MBNL1+rapamycin SSCs were significantly lower than those in DM1-03+Ad-MBNL1 SSCs (Fig. 6c, g, h), whereas there were no significant differences between DM1-03+Ad-MBNL1+rapamycin and DM1-03 SSCs (Fig. 6c, g, h). Similar results were observed in DM1-13-3 SSCs (Fig. 6f, g, h).

These data suggest that MBNL1 promotes proliferation by inhibiting autophagy via the mTOR pathway in DM1 SSCs.

## Discussion

DM1, the most common form of muscular dystrophy affecting adults, is characterized by muscle atrophy, weakness, and myotonia^1^. SSCs, which play an important role in muscle regeneration^6^, can be activated into a proliferating state, and eventually differentiate and fuse with each other, restoring skeletal muscle fiber regeneration ability^35,36^. In this study, MBNL1 was shown to enhance the proliferation of DM1-03 and DM1-13-3 SSCs by inhibiting autophagy activation via the mTOR pathway.

SSCs express the muscle-specific transcription factor MyoD only when they are activated into a proliferating state^7^, but constitutively express Pax7, regardless of their activation state. Here, the proportion of activated SSCs (Pax7^+^MyoD^+^) in the DM1-03 group was lower than that in DM1-04 and DM1-13-3 groups, indicating that DM1-03 SSCs exhibited proliferation defects. In addition, similar results were observed in DM1 SSCs with Ki67 immunofluorescence and CCK-8 assays, consistent with the previous study^9^. However, the proportion of activated SSCs (Pax7^+^MyoD^+^), Ki67-positive cells, and OD 450 values in the DM1-13-3 group were higher than those in the DM1-03 group, but lower than those in the DM1-04 group, indicating that TALEN genome modification targeting CTG repeats promoted the proliferation of DM1 SSCs, but could not restore the proliferation level of normal human SSCs (DM1-04). Although genome modification has been widely used to correct repeat expansion mutations including DM1, it is still a challenge^31,34,37,38^.

MBNL1 dysfunction is reportedly a key event in DM1^10^. In this study, total MBNL1 protein levels were decreased in DM1-03 SSCs and DM1 primary SSCs. However, these reduced levels were ameliorated in DM1-13-3 SSCs, which exhibited enhanced proliferation compared with DM1-03 SSCs. Previous studies have also shown that MBNL1 overexpression can abate the DM1 symptoms^39^. Both low expression and nuclear retention of MBNL1 play an adverse role in the pathogenesis of DM1; therefore, reducing or eliminating the nuclear retention of MBNL1 could be another effective treatment for DM1^13,39^. In this study, we observed nuclear RNA foci in DM1-03 SSCs but not in DM1-13-3 SSCs via RNA fluorescence in situ hybridization. Cytoplasmic MBNL1 expression was elevated in DM1-13-3 SSCs, which exhibited enhanced proliferation compared with DM1-03 SSCs. One previous study demonstrated that, consistent with our findings, elimination of CUGexp RNA with RNA-targeting Cas9 can redistribute endogenous MBNL1 between the nucleus and cytoplasm in DM1^40^. Another study showed that MBNL1 in the cytoplasm of DM1 neurons is in a ubiquitinated state and promotes neurite outgrowth, while MBNL1 in a deubiquitinated state is transported into the nucleus and damages neurite outgrowth^41^. Protein ubiquitination is an ATP-dependent process^42–44^. However, mitochondrial dysfunction and metabolic disorders were observed in DM1 cells^45,46^. ATP deficiency may contribute to the reduction in the level of ubiquitination of MBNL1 and affect its localization in DM1 cells. Taken together, the literature shows that TALEN genome modification not only eliminates toxic RNA and increases total and cytoplasmic MBNL1, but also enhances the proliferation ability of SSCs. Hence, MBNL1 may regulate the proliferation of DM1 SSCs. To further investigate the role of the MBNL1 protein in the proliferation of SSCs, MBNL1 was overexpressed in DM1 SSCs. As expected, MBNL1 overexpression significantly enhanced the proliferative capacity of DM1 SSCs, but was still lower than normal, which indicates that, while MBNL1 could promote the proliferation of DM1 SSCs, it may not be the only regulatory factor. A previous study demonstrated that CUGBP1, another RNA-binding protein whose expression is affected by repeat expansion mutations, can affect the expression of the cell-cycle inhibitor p21 in DM1^47^.

Autophagy is a catabolic and material recycling process, providing raw materials for the intracellular anabolism of cells^18^. mTOR is an important regulatory target for autophagy^48,49^. Downstream cascade autophagy is activated when phosphorylated mTOR is reduced^18^. LC3 (LC3I) in the cytoplasm is converted into LC3II and recruited to the autophagosome membrane. Autophagosomes and lysosomes fuse to form autolysosomes, and the substances within them, including LC3II, are degraded by lysosomal hydrolases. P62/SQSTM1, an autophagy adaptor protein that can interact with LC3 through the LIR domain, is also degraded in the autophagy–lysosome pathway^50^. This autophagy–lysosomal-mediated protein-degradation process can provide sufficient amino acid substrates for cell survival and proliferation^48,49,51^. However, autophagy acts as a double-edged sword in various pathophysiological states^52^. Normal autophagy can maintain cell homeostasis, but disordered autophagy will be accompanied by pathological phenomena, including muscle atrophy^24,53^. One recent study showed that autophagy can degrade cyclin D1 (CCND1) and arrest the cell cycle in the G1 phase to inhibit the proliferation of hepatocellular carcinoma cells^54^. Another study showed that lower-than-normal autophagy levels exert protective effects; however, elevated autophagy levels exert adverse effects^55^. In this study, autophagy levels in DM1 SSCs were elevated, with increased levels of LC3II/LC3I and decreased levels of P62. Similar results were observed in DM1 primary SSCs. Consistent with our findings, previous studies have shown that autophagy levels are increased in DM1^9,27^. Autophagosomes and autolysosomes detected by GFP-mRFP-LC3 significantly increased in DM1 SSCs, indicating activation of the autophagy–lysosomal pathway. Both TALEN genome modification and MBNL1 overexpression significantly reduced autophagic flux in DM1 SSCs. However, TALEN genome modification cannot fully restore autophagy levels in DM1 SSCs. Furthermore, MBNL1 increases the level of phosphorylated mTOR in DM1 SSCs. These data show that MBNL1 may promote the proliferation of SSCs by affecting autophagy via the mTOR pathway.

Next, we investigated the relationship between autophagy and proliferation in DM1 SSCs. Previous studies have shown that inhibiting elevated autophagy levels can promote cell proliferation^24,26^. mTOR overexpression consistently inhibits autophagy levels and promotes the proliferation of DM1 SSCs. PCNA is a eukaryotic protein found in the nucleus of dividing cells, and is essential for DNA replication, excision, and mismatch repair^56^. The expression of proliferation indicator PCNA in DM1 SSCs increased after mTOR overexpression, indicating that inhibiting elevated autophagy levels may promote cell division. However, other studies have reported findings inconsistent with our observations^57^. These differences in outcomes might be because the studies were conducted on different diseases and with different experimental models, cell types, etc. Another critical reason for the observed differences is that autophagy acts as a double-edged sword in various pathophysiological states^52^. Furthermore, the expression of P62 in DM1 SSCs increased after mTOR overexpression, but was still lower than DM1-04 SSCs. Research reports that the level of P62 is reduced in spinal and bulbar muscular atrophy (SBMA)^58^, and the complex restoration of P62 requires autophagic degradation, upregulation of transcription, and amino acid availability^59^. In summary, inhibition of autophagy activation promoted the proliferation of DM1 SSCs. Targeting autophagy and balancing autophagic activity may be valuable strategies for DM1 treatment.

Rapamycin is an mTOR inhibitor^60^. To investigate whether MBNL1 could enhance the proliferation of SSCs by suppressing autophagy via the mTOR pathway, we used rapamycin to target mTOR. Inhibition of the mTOR pathway with rapamycin abolished the enhancement of proliferative capacity mediated by MBNL1 overexpression in DM1 SSCs. Some studies have shown similar results^17^. Therefore, we conclude that MBNL1 can promote the proliferative capacity of DM1 SSCs by suppressing autophagy via the mTOR pathway.

However, there are several issues that need further study. In this study, TALEN genome modification failed to fully restore proliferative capacity in DM1 SSCs. The shortcomings of TALEN genome modification in stem cell therapy have been reflected in previous studies^34,61^. We inserted the PolyA signal for intron 9 to eliminate nuclear RNA foci with the production of truncated DMPK protein^61^. DMPK is a serine/threonine protein kinase, but its biological significance is rarely reported. Its homologous family molecule Rho-associated kinase (ROCK), which controls cell proliferation by regulating cell division, may provide a reference for our research. When a cell divides, the formation of an actomyosin-based contraction loop is important; it can drive the division groove into the cell and then divide it into two daughter cells^62^. Both ROCKs and DMPK can control the contraction of actomyosin by phosphorylating myosin-regulatory light chain (MLC)^63,64^. Truncated DMPK protein may lose or have weakened functions, including the function of phosphorylation of MLC, which needs further research to verify. Another problem is how MBNL1 upregulates the mTOR pathway in DM1 SSCs. Calcineurin is a calcium-dependent serine/threonine phosphatase composed of the catalytic subunit (CnA) and regulatory subunit (CnB) that plays an important role in many pathophysiological events^65^. CnAβ1 is an isoform of CnA that can be upregulated by MBNL1^66^, and promotes the proliferation of myoblasts^67^. Furthermore, CnAβ1 regulates proliferation by sensing metabolic cues to maintain homeostasis in stem cells^68^. It can also reduce catabolism while activating AKT to promote proliferation through the mTOR pathway^69,70^. Interestingly, DM1 cells have metabolic disorders^45,46^. Therefore, we propose the following hypothesis: MBNL1 may reduce autophagy–lysosomal-mediated catabolism and promote proliferation through the CnAβ1–mTOR pathway.

Taken together, these data illustrate the proliferation defect of SSCs differentiated from DM1 patient-derived iPSCs, and suggest that genome modification can eliminate nuclear RNA foci and enhance the proliferation of DM1 SSCs. We did not study the roles of CUG RNA repeat expansion and other factors in proliferation and autophagy in detail, but we conclude that MBNL1 reverses the proliferation defect of DM1 SSCs by inhibiting autophagy via the mTOR pathway. We investigated the mechanisms of DM1 SSC proliferation defects from the perspective of targeted modification of DM1 pathogenic genes and protein molecular pathways. We believe that our findings can provide guidance for the clinical treatment of muscle atrophy in DM1 patients.

## Materials and methods

### Cell lines, maintenance, and differentiation

The normal control DM1-04 iPSC line, the DM1-03 iPSC line, and the DM1-13-3 iPSC line used in this study were in line with the requirements of the University of Florida’s Institutional Review Committee^33,34^.

We used the TeSR^TM^-E8^TM^ Kit (no. 05990, STEMCELL Technologies, Vancouver, BC, Canada) to culture iPSCs in six-well plates coated with Matrigel (no. 354277, Corning Life Sciences, NY, USA). When the monolayers reached 70% confluency, we digested the cells with trypsin at 37 °C for 7 min and plated the single-cell suspensions on a new Matrigel-coated six-well plate. We changed the TeSR^TM^-E8^TM^ medium every day until the cells again reached 70% confluency, and then pretreated the iPSCs with 10 µM Rocki (no. Y-27632, STEMCELL Technologies) in TeSR^TM^-E8^TM^ for 2 h. After Rocki treatment, we discarded the medium and washed the iPSCs with calcium- and magnesium-free DPBS, digested the cells with trypsin at 37 °C for 7 min, and plated them at a density of ~3.0 × 10^5^ iPSCs per well in a new Matrigel-coated six-well plate. When the cultures reached 20% confluency, we initiated differentiation using the sequential culture media reported in our previous study to regulate specific signaling pathways, particularly the Wnt and BMP signaling pathways^44^. On day 25, SSCs were harvested with 0.05% trypsin and replated in Matrigel-coated 24-well plates in Skeletal Muscle Cell Growth Medium (SKGM-2, no. CC-3245, Lonza, Basel, Switzerland). Paired box protein Pax7 (no. AB528428, Developmental Studies Hybridoma Bank, Iowa City, Iowa, USA) levels were monitored by immunofluorescence.

### Open-muscle biopsy

Three DM1 patients (one male and two females, age 46 ± 2 years, means ± SD) with muscular dystrophy symptoms admitted from the First Affiliated Hospital of Chongqing Medical University and three recruited healthy volunteers without underlying disease (two males and one female, age 48 ± 3 years, means ± SD) were included in this study. Muscle biopsies were obtained with the informed consent of the donors. The experimental protocol in this study was approved by the Ethics Committee of the First Affiliated Hospital of Chongqing Medical University. The collected muscle biopsy (quadriceps, ~3 g) was placed in cold PBS supplemented with 1% PeSt (100 U/ml penicillin and 100 µg/ml streptomycin) (no. 15140148, Gibco, Grand Island, NY, USA) and immediately sent to the laboratory.

### Isolation and culture of primary cells

The skeletal muscle of the biopsy was dissected, and the fat tissue and connective tissue visible to the naked eye were removed, minced into small pieces, transferred to the digestion fluid described in a previous study^71^, slowly stirred, and digested at 37 °C for 20 min. The supernatant produced by the digestion was collected and put into the growth medium (10% FBS [no. 16140071, Gibco] and 1% PeSt [no. 15140148, Gibco] in Ham’s F-10 Nutrient Mixture [F-10] [no. 11550043, Gibco]). To obtain enough cells, the undigested tissue was digested once more, and the supernatant was collected. The supernatant obtained from the two digestions was centrifuged at 350 rpm for 10 min, the supernatant was removed, and the cell pellet was resuspended with Ham’s F-10 and plated on a noncoated disk for 1 h to purify SSCs. Then, the supernatant containing purified SSCs was transferred to the coated dish to continue culturing.

### Ki67 and Pax7 immunofluorescence and RNA fluorescence in situ hybridization

Ki67 is a nuclear protein that is expressed in G1, S, G2, and early mitosis cells, but not in G0-phase cells^72^; usually, the ratio of Ki67-positive cells can be used to assess the proliferation ability of SSCs^73^. For staining of cultured SSCs, cells were fixed in 4% PFA for 10 min at room temperature. After the cells were washed in PBS, they were blocked in 10% donkey serum for 1 h at 37 °C and then incubated overnight at 4 °C with primary antibodies targeting Pax7 (1:100, no. AB528428, Developmental Studies Hybridoma Bank), MyoD (1:200, no. PA5-23078, Invitrogen, Carlsbad, CA, USA), and Ki67 (1:100, no. ab15580, Abcam, Cambridge, MA, USA). After three washes in PBS, the cells were incubated with secondary antibodies conjugated with Alexa Fluor Plus 488 (1:300, no. A32766, Invitrogen), Alexa Fluor 555 (1:400, no. A-31572, Invitrogen), and Alexa Fluor 488 (1:300, no. A-21206, Invitrogen) for 1 h at 37 °C. Finally, the cells were washed three times in PBS and stained with DAPI (no. C1005, Beyotime, Shanghai, China). RNA fluorescence in situ hybridization was performed with a Cy5-labeled (CAG)10 DNA probe (GenePharma, Shanghai, China), as described previously^33^. Images were captured with a Nikon AL90 laser confocal scanning microscope (A1+R, Nikon, Tokyo, Japan) and analyzed with ImageJ software.

### Western blot analysis

The cultured cells were homogenized in radioimmunoprecipitation assay (RIPA) lysis buffer (no. P0013B, Beyotime) containing phenylmethanesulfonyl fluoride (PMSF, no. ST505, Beyotime) and phosphatase inhibitors (no. P1045, Beyotime). After the lysates were centrifuged for 15 min, the supernatant was collected for total protein detection. To extract nuclear and cytoplasmic proteins, we used the Nuclear and Cytoplasmic Protein Extraction Kit (no. P0027, Beyotime) according to the manufacturer’s instructions. The protein concentrations were measured using bicinchoninic acid (BCA) protein assay reagent (no. P0010, Beyotime), after which the proteins were loaded into gels and separated by SDS-polyacrylamide gel electrophoresis. After separation, the proteins were transferred to polyvinylidene difluoride (PVDF) membranes (Millipore, Billerica, MA, USA), which were blocked in TBST with 5% nonfat dry milk (no. 100-04504, Bio-Rad, Hercules, CA, USA) or bovine serum albumin for 1 h at room temperature according to the manufacturer’s instructions for primary antibodies. After they were blocked, the membranes were incubated overnight at 4 °C with the following primary antibodies: anti-Pax7 (1:500, no. AB528428, Developmental Studies Hybridoma Bank), anti-PCNA (1:1000, no. sc-56, Santa Cruz Biotechnology, CA, USA), anti-LC3 (1:1000, no. L8918, Sigma Aldrich, St. Louis, MO, USA), anti-P62 (1:1000, no. 5114, Cell Signaling Technology, Danvers, MA, USA), anti-mTOR (1:1000, no. 2983, Cell Signaling Technology), anti-p-mTOR (1:1000, no. 2971, Cell Signaling Technology), anti-MBNL1 (1:1000, no. sc-47740, Santa Cruz Biotechnology), and anti-tubulin (1:1000, no. ab6160, Abcam). After incubation with primary antibodies, the membranes were washed with TBST and incubated with goat anti-mouse IgG (H+L), HRP-conjugate antibody (1:2000, no. SA00001-1, Proteintech, Chicago, IL, USA), goat anti-rabbit IgG antibody (1:3000, no. SA00001-2, Proteintech), or goat anti-rat IgG antibody (1:3000, no. SA00001-15, Proteintech) for 1 h at room temperature. Images were captured with a Fusion FX5 image analysis system (Vilber Lourmat, no. F-77601 Marne-la-Vallée Cedex 3, France).

### mRFP-GFP-LC3

DM1-03 and DM1-13-3 SSCs treated with recommended 10 nM Bafilomycin A1^74^ (no. B1793, Sigma), were used as control groups. SSCs cultured on coverslips were treated with Skeletal Muscle Cell Growth Medium containing adenovirus with the mRFP-GFP-LC3 vector (multiplicity of infection [MOI] = 40, 10^10^ PFU/ml, Hanbio, Shanghai, China) for 2 h; after incubation, the treatment medium was discarded and replaced with regular medium, and the cells were cultured for another 48 h. After adenovirus transduction, the cells were washed with PBS and fixed with 4% paraformaldehyde for 10 min at room temperature. After the cells were washed three times in PBS, they were stained with DAPI (C1005, Beyotime). Images were captured and analyzed with a Nikon AL90 laser confocal scanning microscope (A1+R, Nikon, Tokyo, Japan). Upon transfection of GFP-mRFP-LC3, in the case of autolysosomes, the fluorescence of GFP is quenched so that the observed fluorescence is more red than green. In the case of mRFP, there will always be the same signal strength. In the case of observing more autophagosomes, it would be a nonacidic environment, and therefore, the observed fluorescence would be more yellowish (GFP (GREEN)+mRFP (RED)). A ratio (GFP/mRFP)/cell can quantify what has been broken out of GFP and normalized to the number of cells. The yellow dots after the merging of the red and green fluorescent channels are autophagosomes, and the free red dots indicate autophagolysosomes. ImageJ software was used to count the green and red fluorescent dots and normalize them to the number of cells according to guidelines in previous studies^75,76^.

### Adenovirus infection and drug treatment

To overexpress MBNL1, DM1-03 and DM1-13-3 were cultured in Skeletal Muscle Cell Growth Medium containing an MBNL1-expressing adenovirus (Ad-MBNL1, MOI = 40, 10^10^ PFU/ml, FulenGen, Guangzhou, China) or an empty carrier adenovirus (Ad-control, MOI = 40, 10^10^ PFU/ml, FulenGen, Guangzhou, China) for 2 h. Then, the virus-containing medium was replaced with regular medium, and the cells were cultured for another 36 h.

After MBNL1 overexpression, DM1-03 and DM1-13-3 were serum-starved for 24 h before being treated with 50 nM rapamycin (no. 1292/1, Tocris, Bristol, UK), as recommended in previous publications^77^.

To overexpress mTOR, DM1-03 and DM1-13-3 were cultured in Skeletal Muscle Cell Growth Medium containing an adenovirus-expressing mTOR (Ad-mTOR, MOI = 40, 10^10^ PFU/ml, FulenGen, Guangzhou, China) or Ad-control (MOI = 40, 10^10^ PFU/ml, FulenGen) for 2 h. Then, the virus-containing medium was replaced with regular medium, and the cells were cultured for another 36 h.

### Real-time quantitative reverse transcription polymerase chain reaction (qRT-PCR)

Total RNA from SSCs was extracted with TRIzol (no. 15596018, Invitrogen), and cDNA was synthesized using a PrimeScript^TM^ RT Reagent Kit (no. RR037B, Takara Biotechnology). A Q5 Gradient Real-Time PCR Detection System (Bio-Rad, Hercules, CA, USA) and a TB Green Premix Ex Taq II (no. RR820B, Takara Biotechnology) were used to conduct real-time qRT-PCR under the following reaction conditions: initial denaturation at 95 °C for 30 s followed by 40 cycles of 5 s at 95 °C, 30 s at 60 °C. The primer sequences are listed in Table 1.

**Table 1:** Primers used for qRT-PCR.

Gene	Forward	Reverse
MBNL1	5′-CTCAGTCGGCTGTCAAATCA-3′	5′-AACTGGTGGGAGAAATGCTG -3′
mTOR GAPDH	5′-CTGGGACTCAAATGTGTGCAGTTC-3′ 5′-TGTTGCCATCAATGACCCCTT-3′	5′-GAACAATAGGGTGAATGATCCGGG-3′ 5′-CTCCACGACGTACTCAGCG-3′

### Cell-counting Kit-8 (CCK-8) assay

SSCs were seeded in 96-well plates (3 × 10^3^ cells/well) and cultured in Skeletal Muscle Cell Growth Medium at 37 °C in 5% CO_2_ for 24 h. Then, we added 10 µl of CCK-8 solution (no. C0038, Beyotime) to each well, incubated the cells for 1 h, and immediately measured the absorbance (optical density, OD) at 450 nm with a microplate reader (MB-530, Heales, Shenzhen, China).

### Statistical analysis

SPSS 23.0 was used to analyze the data. ImageJ was used to count cells with positive immunofluorescence, and GraphPad Prism 7.0 was used to create the graphs. Student’s *t* test was used to compare means between two groups; one-way or two-way ANOVA with Bonferroni post hoc test was used to compare means among multiple groups. Data are proven by three independent experiments and presented as the means ± standard deviation (SD). *P* < 0.05 was considered to indicate statistical significance.
